# Antibacterial Activity of a Cationic Antimicrobial Peptide against Multidrug-Resistant Gram-Negative Clinical Isolates and Their Potential Molecular Targets

**DOI:** 10.3390/molecules25215035

**Published:** 2020-10-30

**Authors:** Sandra Patricia Rivera-Sánchez, Helen Astrid Agudelo-Góngora, José Oñate-Garzón, Liliana Janeth Flórez-Elvira, Adriana Correa, Paola Andrea Londoño, Juan David Londoño-Mosquera, Alberto Aragón-Muriel, Dorian Polo-Cerón, Iván Darío Ocampo-Ibáñez

**Affiliations:** 1 Research Group of Microbiology, Industry and Environment, Faculty of Basic Sciences, Universidad Santiago de Cali, Cali 760035, Colombia; helen.agudelo00@usc.edu.co (H.A.A.-G.); adriana.correa00@usc.edu.co (A.C.); 2 Research Group of Chemical and Biotechnology, Faculty of Basic Sciences, Universidad Santiago de Cali, Cali 760035, Colombia; jose.onate00@usc.edu.co; 3 Department of Public Health and Community Medicine, Universidad Icesi, Cali 760032, Colombia; liliana.florez1@correo.icesi.edu.co; 4 Laboratorio de Salud Pública Departamental, Secretaria Departamental de Salud del Valle del Cauca, Gobernación del Valle del Cauca, Cali 760045, Colombia; palondono@valledelcauca.gov.co; 5 Laboratorio de Investigación en Catálisis y Procesos (LICAP), Departamento de Química, Facultad de Ciencias Naturales y Exactas, Universidad del Valle, Cali 760001, Colombia; david.londono@correounivalle.edu.co (J.D.L.-M.); alberto.aragon@correounivalle.edu.co (A.A.-M.); dorian.polo@correounivalle.edu.co (D.P.-C.)

**Keywords:** cationic antimicrobial peptide, multidrug-resistant *Klebsiella pneumoniae*, multidrug-resistant *Pseudomonas aeruginosa*

## Abstract

Antimicrobial resistance reduces the efficacy of antibiotics. Infections caused by multidrug-resistant (MDR), Gram-negative bacterial strains, such as *Klebsiella pneumoniae* (MDRKp) and *Pseudomonas aeruginosa* (MDRPa), are a serious threat to global health. However, cationic antimicrobial peptides (CAMPs) are promising as an alternative therapeutic strategy against MDR strains. In this study, the inhibitory activity of a cationic peptide, derived from cecropin D-like (ΔM2), against MDRKp and MDRPa clinical isolates, and its interaction with membrane models and bacterial genomic DNA were evaluated. In vitro antibacterial activity was determined using the broth microdilution test, whereas interactions with lipids and DNA were studied by differential scanning calorimetry and electronic absorption, respectively. A strong bactericidal effect of ΔM2 against MDR strains, with minimal inhibitory concentration (MIC) and minimal bactericidal concentrations (MBC) between 4 and 16 μg/mL, was observed. The peptide had a pronounced effect on the thermotropic behavior of the 1,2-dimyristoyl-sn-glycero-3-phosphocholine (DMPC)/1,2-dimyristoyl-sn-glycero-3-phosphorylglycerol (DMPG) membrane models that mimic bacterial membranes. Finally, the interaction between the peptide and genomic DNA (gDNA) showed a hyperchromic effect, which indicates that ΔM2 can denature bacterial DNA strands via the grooves.

## 1. Introduction

Antibiotic resistance is a serious global health concern caused by the ineffectiveness of empirical antibiotic therapy for a wide range of infections caused by Gram-negative bacteria [1]. Several factors, including the excessive use of antibiotics, incomplete course of treatment, and self-medication can accelerate the development of resistant bacterial (RB) strains [1]. Recently, the World Health Organization (WHO) published the first list of priority pathogens resistant to antibiotics. The list includes 12 families of bacteria that pose the greatest threat to human health. Thus, research and development of new antibiotics is urgently needed [2]. This list includes multidrug-resistant (MDR) *Klebsiella pneumoniae* (MDRKp) and *Pseudomonas aeruginosa* (MDRPa), which are resistant to several antibiotics, including carbapenems and third generation cephalosporins [2]. MDR bacteria are resistant to at least one agent in three or more antibiotic categories and circulate in non-hospital and hospital settings, where they can cause nosocomial infections [3,4,5,6].

MDRKp and MDRPa isolates carry resistance genes and are highly resistant to a broad spectrum of antibiotics, including carbapenems, aminoglycosides, fluoroquinolones, and polymyxins [7,8,9,10,11,12,13,14]. An increase in the number of MDRKp and MDRPa strains leads to more cases of persistent infections, which increases the morbidity and mortality of hospitalized or immunocompromised individuals worldwide [9,13,14,15,16,17,18,19]. The emergence of MDR *K. pneumoniae* and *P. aeruginosa* strains is due to the acquisition of resistance genes combined with the presence of mechanisms of resistance to multiple antimicrobials [20]. These strains acquire resistance through chromosomal gene mutations and the transfer of different mobile genetic elements to plasmids, which move between the cells of different species [11]. Carbapenem-resistant MDRPa and MDRKp clinical isolates, mediated by *bla_KPC_, bla_VIM_*, *bla_IMP_*, *bla_NDM_,* and *bla_OXA-48_,* and *bla_KPC_* and *bla_VIM_* are found circulating in Colombia [14,17].

Circulation of MDR clinical isolates is a public health concern, as infections caused by MDR strains are difficult to treat due to their reduced susceptibility to conventional antimicrobial agents [7,12]. Therefore, alternative therapeutic strategies for treating infections caused by MDR bacteria are urgently needed [7]. Antimicrobial peptides (AMPs) have emerged as an alternative to control resistant bacteria owing to their broad-spectrum activity, high-efficacy at low concentrations, target-specificity, low propensity to resistance, and synergistic action with classic antibiotics [21]. AMPs are a large group of naturally occurring antimicrobials identified in the innate immune system of several species, from plants to humans [22,23,24,25,26]. In particular, cationic AMPs (CAMPs) have emerged as a promising alternative against MDR bacteria because they are highly effective in killing RB strains, compared to conventional antibiotics [7,26,27,28,29,30,31,32,33]. CAMPs can display direct activity against diverse cellular targets, by disrupting the integrity of bacterial membranes, nucleic acids, and proteins or inhibiting intracellular functions, including the synthesis of these macromolecules [7,26,27,28,29,30,32]. Several AMPs have an affinity for bacterial membrane phospholipids and nucleic acids, including DNA and RNA [21,34,35,36,37,38]. AMPs damage genomic or plasmid DNA after disrupting bacterial membranes or covalent and non-covalent interactions with DNA [37,38,39,40,41]. Therefore, bacterial membranes and DNA are ideal targets for evaluating AMP activity.

Diverse groups of AMPs are effective against bacteria and other pathogens [25,31,33,42]. Cecropins and cecropin-derived CAMPs are antimicrobial peptides with bactericidal activity against wild-type and MDR bacteria [42,43]. Cecropins belong to a group of naturally occurring AMPs in insects and exhibit in vitro activity against bacteria, including Gram-positive and Gram-negative species [43]. In this study, we investigated the in vitro antibacterial activity of a synthetic CAMP derived from cecropin D-like peptide from *Galleria mellonella* called ΔM2 [44] against clinical strains of *K. pneumoniae* and *P. aeruginosa*. Here we evaluated the effects of CAMP against the wild-type strains of *P. aeruginosa* (WTPa) and *K. pneumoniae* (WTKp) and the clinical isolates of *P. aeruginosa* MDRPa and *K. pneumoniae* MDRKp. Previously, ΔM2 was shown to be effective against Gram-positive and Gram-negative ATTC strains; however, its effect on clinical isolates is unknown [44]. Moreover, analyses of peptide interaction with model membranes mimicking bacterial membranes and DNA from MDR strains were developed to evaluate potential targets of ΔM2 in Gram-negative bacteria.

## 2. Results and Discussion

### 2.1. Clinical Isolates: Antibiotic Susceptibility and Resistance Genes

The antibiotic susceptibility and resistance profiles of *K. pneumoniae* and *P. aeruginosa* clinical isolates are summarized in Table 1 and Table 2, respectively. From 30 *K. pneumoniae* clinical isolates, 15 strains were WTKp and the remaining were classified as MDRKp because they showed resistance to at least three from the six antimicrobial categories used to construct the susceptibility and resistance profiles (Table 1). The MDRKp strains showed resistance to penicillin + β-lactamase inhibitors, cephamycins, extended spectrum cephalosporins, aminoglycosides, fluoroquinolones, and carbapenems (Table 1). In particular, the confirmation of the molecular classes of carbapenemases and results of the Carba NP test showed that two MDRKp strains carried only *bla_KPC_* for class A serine carbapenemase; six MDRKp clinical isolates were metallo-beta-lactamases-producing (MBL) *K. pneumoniae* strain with *bla_NDM_, bla_VIM_* and/or *bla_IMP_*; and seven MDR *K. pneumoniae* strains showed co-production of carbapenemase because they carried a combination of *bla_KPC_*, *bla_NDM_,* and *bla_VIM_* and/or *bla_IMP_* (Table 1). Moreover, from 30 *P. aeruginosa* clinical isolates, 15 were classified as WTPa and 15 as MDRPa, which was resistant to antipseudomonal cephalosporins, fluoroquinolones, penicillin + β-lactamase inhibitors and carbapenems, aminoglycosides, and polymyxins (Table 2). According to molecular characterization, 13 MDRPa carried *bla_NDM_,* and *bla_VIM_* and/or *bla_IMP_*, which encode MBL enzymes; whereas the two remaining MDRPa strains contained a combination of *bla_KPC_*, *bla_NDM_,* and *bla_VIM_* and/or *bla_IMP_*, which indicates the co-production of class A serine carbapenemase and MBL enzymes (Table 2). Thus, the MDRKp and MDRPa strains can be considered “high-risk clones,” similar to those that are endemic and circulate in Colombia, and represent a serious threat for public health [14,17]. The presence of MDRKp and MDRPa isolates is consistent with the bacterial resistance situation in Colombia, where a high frequency of MDR *K. pneumoniae* and *P. aeruginosa* strains carrying carbapenemase genes has been reported [14,17].

### 2.2. Antibacterial Activity of ΔM2 against Clinical Isolates

Several studies have evaluated the antibacterial activity of CAMPs due to their high potency and rapidity in killing bacterial cells [24,26,31,42]. Here, we investigated the in vitro antibacterial activity of ΔM2 against wild-type and MDR clinical isolates of *K. pneumoniae* and *P. aeruginosa*, which is summarized in Table 3. First, the antibacterial activity of ΔM2 against ATTC strains was confirmed and the peptide was evaluated against clinical isolates (Table 3). ΔM2 had antibacterial activity against ATCC strains of *Escherichia coli* and *P. aeruginosa*, with minimal inhibitory concentrations (MIC) values between 4 and 8 μg/mL. (Table 3). The effectiveness of this peptide against ATCC Gram-negative strains was consistent with previous reports in which natural cecropins and/or synthetic cecropin-analogs were effective against laboratory strains of *E. coli* and *P. aeruginosa* [31,43,44,45,46,47,48]. Similar to ATCC strains, the clinical isolates of *K. pneumoniae* and *P. aeruginosa* showed susceptibility to ΔM2, with MIC values from 4 to 16 μg/mL. (Table 3). However, the MIC values found here for clinical isolates were considerably lower in comparison with those reported previously for other cationic peptides, but were similar to those reported for the cecropin A-melittin hybrid peptide [31,42,49].

**Figure 1:**
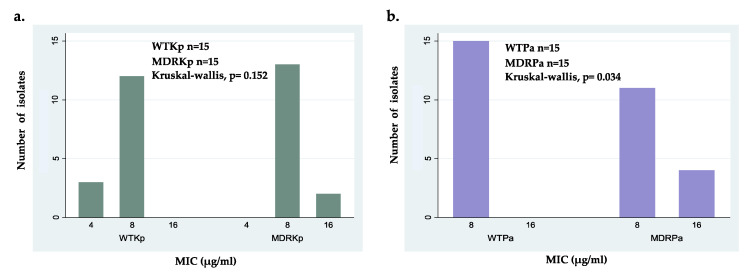
Minimal inhibitory concentration (MIC) distribution of ΔM2 for: (**a**) wild-type isolates (WTPa) and multidrug- resistant isolates (MDRPa) of *Pseudomonas aeruginosa*; (**b**) wild-type isolates (WTKp) and multidrug- resistant isolates (MDRKp) of *Klebsiella pneumoniae.*

Following the comparison of tested isolates, no significant differences were found between wild-type strains of both species, but statistical differences were found between the MDRKp and MDRPa strains (Table 3). Despite these significant differences, ΔM2 exhibited strong activity against all MDR strains, with MIC values between 8 and 16 μg/mL (Table 3 and Figure 1). This efficacy was comparable to that reported previously for other AMPs that have bactericidal activity against MDR strains of Gram-negative bacteria with several resistance mechanisms, including KPC-producing *K. pneumoniae* and colistin-resistant *P. aeruginosa* [49,50,51,52,53]. The statistical difference of the effectiveness of ΔM2 between MDR isolates of *K. pneumoniae* and *P. aeruginosa* can be explained by the presence of virulence factors and resistance mechanisms in strains. For example, KPC-producing *K. pneumoniae* strains have a capsule that contributes to their pathogenicity due to the capsular polysaccharides that protect them from the antibacterial activity of ΔM2 [12,41,54,55]. Moreover, the mechanisms and systems associated with colistin resistance in some MDRPa strains can modulate the efficiency of ΔM2 [49]. We found statistical differences in MICs when colistin-susceptible MDR *P. aeruginosa* and colistin-resistant MDRPa clinical isolates were compared (*p* = 0.0011) (Table 2), which suggests that colistin cross-resistance can modulate ΔM2 effectiveness. However, further investigations are necessary in the future.

Intraspecific comparisons showed no significant differences between WTKp and MDRKp strains; however, the wild-type clinical isolates showed higher susceptibility with the lowest MIC value of 4 μg/mL (Figure 1a). Interestingly, these findings suggest that the activity of ΔM2 against *K. pneumoniae* is independent of its antibiotic resistance pattern (Figure 1a). In contrast, statistical differences were found when susceptible and MDR strains of *P. aeruginosa* were compared, which can be explained by the several resistance mechanisms in some MDRPa strains that modulate the effectiveness of ΔM2 (Figure 1b). In fact, several MDR *P. aeruginosa* clinical isolates showed slightly lower susceptibility to ΔM2, with MICs of 16 μg/mL, compared to WTPa strains (Figure 1b). Because no differences were found between the MICs and MBCs of ΔM2 against all clinical isolates tested in this study, this peptide may be considered a bactericidal agent (Table 3). These findings showed that ΔM2 is a bactericidal peptide active against the clinical isolates of *K. pneumoniae* and *P. aeruginosa*, including wild-type and MDR strains. The bactericidal effect of several AMPs, including cecropin-derived peptides, cathelicidins, magainins, and nisins, against MDR strains has also been reported [30,31,33,42,49,56].

Although ΔM2 showed slight hemolytic activity in a previous study [44], its therapeutic index (TI) in Gram-negative bacteria was above 128. Taking this and the results obtained into account, ΔM2 may be safe as a topical formulation [57] to treat infectious diseases caused by MDR bacteria.

### 2.3. Interaction of ΔM2 with Potential Bacterial Targets

#### 2.3.1. Interaction of ΔM2 with Model Bacterial Membrane

**Figure 2:**
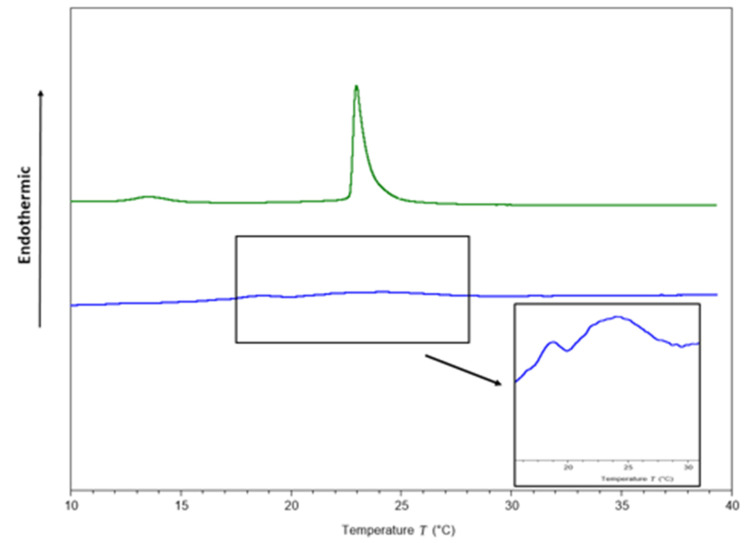
Thermograms of DMPC/DMPG (3:1) multilamellar vesicles (MLVs) in the absence (green line) and presence of the peptide (blue line) at a peptide–lipid molar ratio 1:10.

To identify potential targets of ΔM2 in Gram-negative bacteria, studies on the interaction with model membranes mimicking bacterial membranes were performed. Two endothermic peaks (Figure 2), one for pre-transition at 13.55 and other for main transition at 22.98 °C, were obtained in the thermograms from membrane models consisting of pure 1,2-dimyristoyl-sn-glycero-3-phosphocholine (DMPC)/1,2-dimyristoyl-sn-glycero-3-phosphorylglycerol(DMPG) phospholipids (Table 4). The transition temperature from gel to crystalline liquid is concordant with that reported by Aragón-Muriel et al. [58]. In contrast, the full width at half maximum of the peak (FWHM) was 0.55 °C (Table 4).

After adding the peptide at a 1:10 peptide–lipid molar ratio, a pronounced effect was observed on the thermotropic behavior of the DMPC/DMPG membranes (Figure 2). The pre-transition peak, which is sensitive to foreign molecules [59], was completely abolished, and the main transition peak visually disappeared (Figure 2). However, for scaling the Y axis, the formation of two phases was observed (Figure 2), one more fluid than the other, in accordance with a previous report conducted by Oñate-Garzón in 2017, where a similar behavior was observed at 1:25 peptide–lipid ratio [44].

The cationic charge of the peptide could be involved in membrane disturbance, due to the presence of the anionic DMPG in the phospholipids mixture, as the cationic arginine side chain can approach 1,2-dimyristoyl-sn-glycero-3-phosphorylglycerol (DMPG) head groups to 5 Å [60]. Table 4 shows that the transition enthalpy was reduced from 0.32 to 0.03 cal/g after the peptide was added, suggesting that less heat is needed to achieve phase transition because the peptide disrupts interactions between lipid acyl chains, as a result of the interruption of the intra and intermolecular interactions of van der Waals and trans-gauche isomerization [44]. Additionally, FWHM increased considerably from 0.5 to 3.69 °C as a consequence of the interaction between the peptide with the membranes, suggesting a decrease in cooperativity.

#### 2.3.2. Interaction of ΔM2 with Bacterial DNA

AMPs use different modes of action for killing bacteria, including membrane disruption, interaction with intracellular molecules, such as DNA, RNA, or proteins, and influencing biochemical processes that are vital for bacterial survival [7,26,27,28,29,30,32]. To explore the genomic DNA as a potential target for ΔM2 in Gram-negative bacteria, interaction assays between peptide and bacterial DNA were performed. These analyses could give insights into the mechanisms of action of ΔM2.

##### Electronic Absorption Spectra

**Figure 3:**
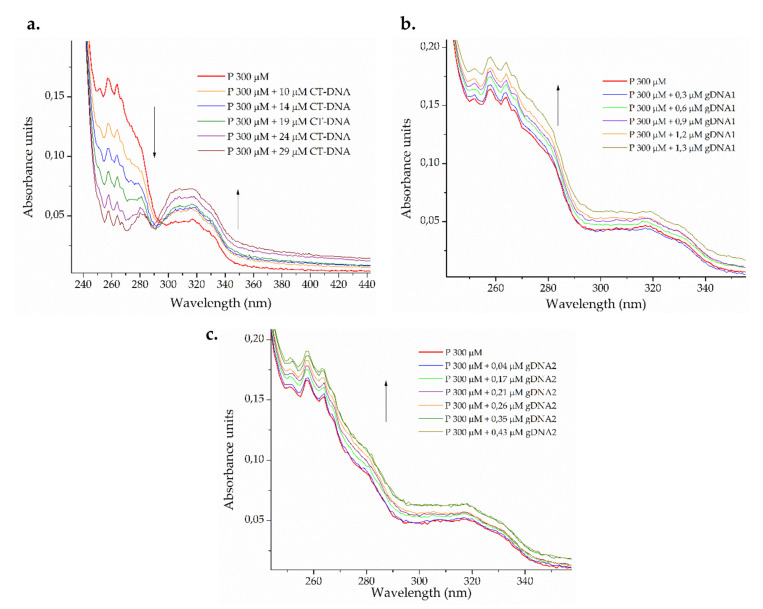
Photometric titration of 300 μM ΔM2 peptide (P) with (**a**) eukaryotic calf thymus DNA (CT-DNA) and genomic DNA from (**b**) susceptible *Pseudomonas aeruginosa* ATCC 27853 (gDNA1) and (**c**) resistant *Klebsiella pneumoniae* ATCC 2146 (gDNA2).

DNA interaction studies were carried out by monitoring the changes on the electronic absorption spectra of bacterial genomic DNA and calf thymus DNA (CT-DNA) with peptide titration. The effect on the peptide spectrum after the addition of different amounts of CT-DNA and bacterial genomic DNA from susceptible *P. aeruginosa* ATCC 27853 (gDNA1) and resistant *K. pneumoniae* ATCC 2146 (gDNA2) is shown in Figure 3.

A dual effect on the electronic spectrum of the peptide (Figure 3a) was observed after the addition of increasing amounts of eukaryotic CT-DNA. The isosbestic point was observed around 295 nm, indicating the formation of a third, new chemical entity in the binary system [61]. At wavelengths lower than the isosbestic point, a hypochromic effect was observed, whereas hyperchromic behavior was found at wavelengths longer than the isosbestic point. The peptide may be binding CT-DNA in more than one way, with insertion (intercalative and groove binding mode) being the most probable [62]. For bacterial genomic DNA, smaller peptide-gDNA ratios were used. The hyperchromic effect was observed in both cases (Figure 3b,c), which indicates the capability of denaturing smaller DNA strands via groove interactions [61,63].

**Figure 4:**
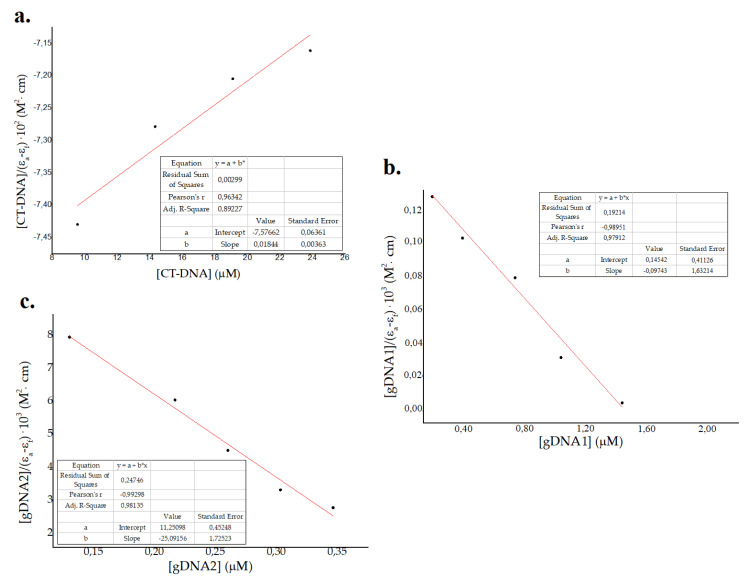
Wolf–Shimer plots for (**a**) eukaryotic calf thymus DNA (CT-DNA) and genomic DNA from (**b**) susceptible *Pseudomonas aeruginosa* ATCC 27853 (gDNA1) and (**c**) resistant *Klebsiella pneumoniae* ATCC 2146 (gDNA2).

Data from photometric titration were collected and fitted to the Wolfe–Shimer Equation (1) for the estimation of binding constants (*Kb*) in eukaryotic and prokaryotic genomic DNAs. This equation was derived from previous thermodynamic and kinetic studies conducted by Schmechel et al. on the intrinsic binding of small molecules with DNA strands [64]3. Equation (1) represents a simplified model where the ratio [DNA]/[Peptide] is low and the intrinsic binding constant *Kb* can be estimated for various small molecules.

(1) $$\frac{\left[\mathrm{DNA}\right]}{\varepsilon_{a}-{\;\varepsilon }_{f}}=\frac{\left[\mathrm{DNA}\right]}{\varepsilon_{b}-{\;\varepsilon }_{f}}+\frac{1}{\left[K_{b}\left(\varepsilon_{b}-{\;\varepsilon }_{f}\right)\right]}$$

where [DNA] refers to DNA concentration and ε_a_, ε_b_ and ε_f_ represent apparent, fully bonded, and free molar extinction coefficients of the peptide, respectively [64,65,66]. The Wolf–Shimer plots, using absorbance of the peptide at 257 nm vs. CT-DNA and gDNA–1gDNA2 concentration, is shown in Figure 4. *Kb* values obtained were 2 × 10^3^ M^−1^, 0.5 × 10 ^6^ M^−1^, and 2 × 10^6^ M^−1^ for eukaryotic CT-DNA, prokaryotic gDNA1, and prokaryotic gDNA2, respectively. These results show a major effect in the prokaryotic genome compared to the eukaryotic genome, which suggests a selectivity of ΔM2 for bacterial DNA [40,41]. Values of classic intercalators and DNA markers are in the order of 10^6^–10^8^ [66,67], which are larger for the CT-DNA binding constant and comparable for gDNA1 and gDNA2. Major binding constants for prokaryotic genomes is attributed to the size and packing properties, in comparison to that in eukaryotic genomes [40,41]. As bacterial DNA is not as well-packaged as eukaryotic DNA, small molecules are likely to interact with it and affect genomic processes at lower concentrations [40,41]. Moreover, the binding constants for both susceptible and resistant bacterial genomes were in the same order of magnitude (Figure 3 and Figure 4). Thus, the interaction between ΔM2 and DNA from Gram-negative bacteria is independent of their antibiotic resistance patterns (Figure 3 and Figure 4).

##### Agarose Gel Electrophoresis

**Figure 5:**
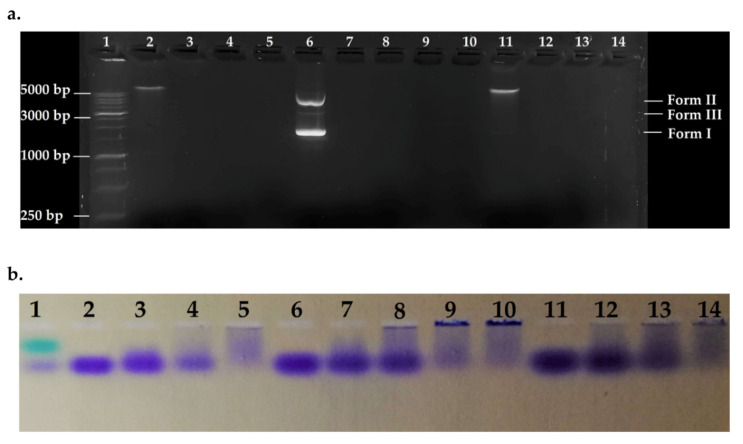
Agarose gel electrophoretic pattern. Lane 1: HyperLadder 1 kb (15 μL); Lane 2: gDNA1; Lane 3: [P]/[gDNA1] = 0.93; Lane 4 = [P]/[gDNA1] = 1.86; Lane 5: [P]/[gDNA1] = 4.65; Lane 6: pmCherry; Lane 7: [P]/[pmCherry] = 0.93; Lane 8: [P]/[pmCherry] = 1.86; Lane 9: [P]/[pmCherry] = 3.1; Lane 10: [P]/[pmCherry] = 4.65; Lane 11: gDNA2; Lane 12: [P]/[gDNA2] = 0.93; Lane 13: [P]/[gDNA2] = 1.86; Lane 14: [P]/[gDNA2] = 4.65. Ratios are calculated using mass concentration. (**a**) Revealed gel; (**b**) Stained gel 1.

To detect the effect of the peptide on bacterial genomic DNA from susceptible and resistant strains, fixed amounts of gDNA1, gDNA2, and pmCherry (as control) were incubated in the presence of variable concentrations of ΔM2 and the systems were electrophoresed. Electrophoretic patterns are shown in Figure 5. Peptide-DNA ratios were fitted to match ratios of different zones in the curves shown in Figure 4. All DNAs were denatured in the evaluated ratios, showing a major effect on bacterial genomes. gDNAs was over 5000 bp and pmCherry vector approximately 2500 bp in size. The phenomenon of DNA aggregation and sedimentation is seen in Figure 5b, as indicated by bromophenol blue loading buffer. Wells with high [P]/[DNA] ratios showed major DNA sedimentation; hence, null mobility and no fluorescent marking (Figure 5a), this confirms the results obtained by UV-vis assays. Thus, denaturation of bacterial DNA may not be via hydrolysis but by non-specific precipitation of a peptide-DNA complex, which cannot run through the gel [68].

Thus, the peptide-DNA interaction may occur by groove binding and electrostatic interaction. The peptide possesses a net positive charge, which neutralizes the negative charge of the phosphate backbone, inducing precipitation in an aqueous environment. The interaction is concentration dependent, as shown in Figure 4, and small bacterial genomes are more affected, as seen by the higher binding constants [68]. No differences were found when the interactions of gDNA1 and gDNA2 with ΔM2 were compared, suggesting that bacterial DNA interacts with the peptide through electrostatic interactions or groove binding (Figure 5). Additionally, these results complemented the electronic absorption analysis and confirmed that the effect of the peptide on bacterial genomic DNA is independent of the strains’ resistance profile and mechanism used.

## 3. Materials and Methods

### 3.1. Peptide Design and Synthesis

ΔM2 is a CAMP derived from the neutral peptide, cecropin D-like, from *G. mellonella*, that has antibacterial activity against laboratory strains [44]. This peptide is composed of 39 residues and has a net charge of +9 at neutral pH. ΔM2 was provided by GenScript Corporation (Piscataway, NJ, USA) at 98% purity. The lyophilized peptide was dissolved in phosphate buffered saline (pH 7.4; 138 mM NaCl, 3 mM KCl, 1.5 mM NaH_2_PO_4_, and 8.1 mM Na_2_HPO_4_), at an initial concentration of 5000 μg/mL. Dilutions were prepared on the day of use.

### 3.2. Identification of Clinical Isolates

A total of 60 clinical strains were tested in this study: 30 each for *K. pneumoniae* and *P. aeruginosa*. All clinical isolates were isolated from clinical specimens, such as urine, secretions, and blood, recovered from two tertiary care hospitals in Cali, Colombia, between 2017 and 2019. All cultures of clinical isolates were sent to Microbiology Laboratory at Laboratorio de Salud Pública Departamental del Valle del Cauca (LSPD-Valle), where bacterial identity was confirmed, and antibiotic susceptibility characterization was performed. Species identification was performed using the automated VITEK^®^ 2 system, (bioMerieux, 9.02, Marcy l’Etoile, France) with the VITEK^®^ 2 Gram-Negative Identification card (VITEK^®^ 2 GN ID), which is based on established biochemical methods and substrates that evaluate the use of carbon, enzymatic activity, and resistance (Ref. 21341, bioMerieux, Marcy l’Etoile, France). All laboratory strains, including *E. coli* ATCC^®^ 25922™, *K. pneumoniae* ATCC^®^ 2146™, and *P. aeruginosa* ATCC^®^ 27853™, were obtained from the American Type Culture Collection (ATCC, Manassas, VA, USA).

### 3.3. Characterization of Clinical Isolates: Resistance Profile and Identification of Resistance Genes

The phenotypical and genotypical characterizations of the resistance of clinical isolates were performed. For phenotypic characterization, the resistance and susceptibility of the bacterial strains were determined using the VITEK^®^ 2 Antimicrobial Susceptibility Testing (VITEK^®^ N272-AST) card in the VITEK^®^2 system (Ref. 414164, bioMerieux) to confirm the in vitro MIC, according to the clinical breakpoints defined by the Clinical Laboratory Standards Institute (CLSI) [69]. The susceptibility and resistance of all *K. pneumoniae* isolates to antimicrobial agents, such as penicillin + β-lactamase inhibitors (ampicillin/sulbactam (SAM) and piperacillin/tazobactam (TZP)); cephamycins (cefoxitin (FOX)); extended-spectrum cephalosporins (ceftazidime (CAZ), ceftriaxone [CRO], and cefepime (FEP)); carbapenems (doripenem (DOR), ertapenem (ETP), imipenem (IPM), and meropenem [MEM]); aminoglycosides (amikacin [AMK] and gentamicin (GEN)), and fluoroquinolones (ciprofloxacin (CIP)), were determined. *E. coli* ATCC^®^ 25922™ and *K. pneumoniae* ATCC^®^ 2146™ strains were used as references for WTKp and MDRKp, respectively. All *P. aeruginosa* isolates were tested against TZP, CAZ, FEP, DOR, IPM, MEM, AMK, GEN, CIP, and polymyxins (colistin (CST)). *P. aeruginosa* ATCC^®^ 27853™ was used as the reference for WTPa. The susceptibility and resistance of ATCC strains were also confirmed using the VITEK^®^ 2 AST card. All colistin-resistant MDRPa clinical isolates were confirmed using the VITEK^®^2 system by the reference broth microdilution method, according to CLSI [70]. Additionally, the resistance mechanisms of clinical isolates that showed resistance to extended spectrum cephalosporins and at least one carbapenem was confirmed by RAPIDEC^®^ CARBA NP (Biomerieux), according to the recommendations of CLSI [69]. Finally, for genotypic characterization, the presence of carbapenemase genes, such as *bla_KPC_, bla_VIM_, bla_IMP_, bla_NDM_*, and *bla_OXA-48_*, was confirmed by the automated rapid real-time PCR assay BD MAX Check-Points CPO (Check-Points, Wageningen, The Netherlands) in the BD MAX^™^ system (Ref. 278102).

### 3.4. Antimicrobial Assay of ΔM2

The MIC values of ΔM2 were determined by the broth microdilution test, according to the protocol of CLSI [69,71]. Briefly, pure clinical isolates were grown in brain heart infusion agar and incubated at 37 °C for 18–24 h. A colony from the pure culture was initially resuspended in sterile water to reach a turbidity value of 0.5 McFarland, and the resulting suspension contained approximately 1–4 × 10^8^ colony forming units (CFU)/mL. Using this suspension, a final solution was obtained with a concentration of 2–7 × 10^5^ CFU/mL in cation-adjusted Mueller–Hinton broth. The bacterial inoculum was incubated with different concentrations of ΔM2. The highest tested concentration of 128 μg/mL was used for serial 1:2 dilutions. In a final volume of 100 μL, peptide and inoculum were incubated in sterile 96-well polypropylene microplates (Sigma-Aldrich, St. Louis, MO, USA) at 37 °C for 18–20 h. A peptide-free control was used for every isolate evaluated. Additionally, the reference ATCC strains were used in each assay as controls to ensure reproducibility. The MIC of ΔM2 for each strain was defined as the lowest concentration that inhibited the visible growth of bacteria after incubation [69,71]. The minimal bactericidal concentrations (MBCs), defined as the lowest concentration of an antibacterial agent required to kill 99.9% of a particular bacterium, were determined by plating the contents of the first three wells showing no visible bacterial growth onto Muller–Hinton agar plates and incubated at 37 °C for 18–20 h [31,72]. MICs and MBCs were determined in duplicate, and at least three independent assays were performed for each isolate.

### 3.5. Interaction of Model Membranes

#### 3.5.1. Membrane Preparation

Dehydrated anionic 1,2-dimyristoyl-sn-glycero-3-phosphorylglycerol (DMPG) and zwitterionic 1,2-dimyristoyl-sn-glycero-3-phosphocholine (DMPC) lipids were dissolved in chloroform/methanol (2:1 v/v). A mixture of DMPC/DMPG (3:1), which mimics bacterial membranes, was dried under a gentle stream of nitrogen, and placed under vacuum for 3 h to remove any residual solvent. The lipid films were hydrated with HEPES buffer (25 mM HEPES, pH 7.0; 100 mM NaCl and 0.2 mM EDTA), vigorously vortexed for 2 min, and incubated for 10 min at 37 °C above the phase transition temperature (Tm) three times to obtain multilamellar vesicles (MLVs) [58].

#### 3.5.2. Differential Scanning Calorimetry

DSC analysis was made using a DSC Q25 (TA instrument, New Castle, DE, USA). MLVs were prepared using 1 mg of lipids to give two peptide–lipid ratios: 1:50 and 1:10. HEPES buffer was used as a reference solution. Samples were encapsulated in standard aluminum DSC pans, and scanning was carried out over a range of 10 °C–40 °C at a heating rate of 1 °C/min. Thermograms were acquired and analyzed using the Trios software package (TA Instruments) to obtain the phase transition temperature (Tm), the transition enthalpy (DH), and the FWHM.

### 3.6. DNA Interaction Assays

DNA interaction studies with the peptide were carried out by electronic absorption experiments and agarose gel electrophoresis. The interaction of ΔM2 with DNA was performed using bacterial genomic DNA from the susceptible *P. aeruginosa* ATCC 27853 strain (gDNA1) and the resistant *K. pneumoniae* ATCC 2146 strain (gDNA2). To evaluate the selectivity of the peptide, a control with highly polymerized, lyophilized calf thymus DNA (CT-DNA) from Sigma-Aldrich was used. Genomic DNA from bacterial stains was extracted using the GenoLyse^®^ kit, according to the manufacturer’s instructions (Hain Lifescience-BRUKER, Nehren, Germany). The pmCherry vector from was extracted from *E. coli* BL21 (DH5*α*) using Kit Hi-Speed Mini Plasmid from IBI Scientific [73]. All DNA solutions had an A_260_/A_280_ value between 1.8 and 1.9, indicating that the DNA was free of RNA and protein. The DNA was resuspended 10 mmol/L Tris and 1 mmol/L EDTA in deionized water, with the pH adjusted to approximately 7.5. The DNA solutions were stored at −5 °C.

#### 3.6.1. Electronic Absorption Monitoring Assays (UV-Vis)

The electronic absorption spectra were recorded in an UV-visible Evolution 220 Thermo Fisher Scientific spectrophotometer (Waltham, MD, USA), equipped with Single Cell Peltier System for temperature control. The interaction between ΔM2 was carried out with gDNA1, gDNA2, and CT-DNA titrations at a constant concentration of the peptide (300 µM) at 25 °C. After the addition of each titrant, the electronic absorption spectra of the evaluated peptide were measured between 230 nm and 450 nm. DNA absorbance was eliminated by adding an equal amount of DNA to the sample and the standard solution, as reported in the literature [49,52]. A CT-DNA solution 11,000 µM in nucleotides were prepared using ε_260_ = 6600 cm^−1^∙mol^−1^∙L as molar extinction coefficient and measuring the absorbance at 260 nm. The stock solution was stored at −5 °C. The electronic absorption experiments were determined in duplicate, and at least three independent assays were performed for each DNA.

#### 3.6.2. Agarose Gel Electrophoresis

The interaction between ΔM2 and bacterial genomic DNA was studied by agarose gel electrophoresis. Mixtures of the peptide at different concentrations, with gDNA1 and gDNA2 at constant concentrations, in a final volume of 20 μL were incubated in the Eppendorf ThermoMixer^®^ C under physiological conditions (37 °C and pH = 7.0) for 90 min. The pmCherry vector extracted from *E. coli* BL21(DH5*α*) was used as a control. After the incubation was complete, the samples were loaded in 1% agarose gel with 1× TAE buffer and Bioline HyperLadder™ 1 kb as the molecular weight marker. The Owl™ EasyCast™ B1 Mini Gel electrophoresis system was used for electrophoresis. GelGreen™ (GoldBio, St. Louis, MO, USA) was used to visualize DNA on a MaestroGen UltraBright Led 470 nm transilluminator (Hsinchu, Taiwan).

### 3.7. Statistical Analysis

The results were analyzed using descriptive statistical tools with the median. Statistically significant differences in MICs were analyzed and compared using the non-parametric Kruskal–Wallis test with R-Project software Version 1.1.463. *p*-values ≤ 0.05 were considered statistically significant.

## 4. Conclusions

In this study, we have demonstrated that ΔM2 exhibits strong bactericidal activity against both susceptible and MDR clinical isolates of *K*. *pneumoniae* and *P*. *aeruginosa.* However, significant differences were found between the MDRKp and MDRPa clinical isolates, possibly due to differences in resistance mechanisms between *K*. *pneumoniae* and *P*. *aeruginosa,* which can modulate ΔM2 effectiveness. Further investigation is warranted in the future. The ΔM2 peptide interacted markedly with the model membrane that mimics bacterial membranes and bacterial genomic DNA from susceptible and resistant strains, thereby providing insights into the mode of action of ΔM2 in interacting with potential molecular targets in Gram-negative bacteria. Considering these results and the TI in Gram-negative bacteria for ΔM2, this peptide may be safe as a topical formulation to treat infectious diseases caused by MDR bacteria.

## Figures and Tables

**Table 1:** Antimicrobial susceptibility and resistance profiles of wild-type and multidrug-resistant strains of *K. pneumoniae.*

Strain ID	Carbapenemase Gene	MIC (μg/mL) for Antibiotic/Interpretative Categories													Carba NP Result
		SAM ^1^	TZP ^1^	FOX ^2^	CAZ ^3^	CRO ^3^	FEP ^3^	DOR ^4^	ETP ^4^	IPM ^4^	MEM ^4^	AMK ^5^	GEN ^5^	CIP ^6^	
Kp 01	*bla_NDM_,* and *bla_VIM_* and/or *bla_IMP_*	≥32/R	≥128/R	≥64/R	≥64/R	≥64/R	≥64/R	≥8/R	≥8/R	≥16/R	≥16/R	32/I	≥16/R	=4/R	POS
Kp 02	*bla_NDM_*	≥32/R	≥128/R	≥64/R	≥64/R	≥64/R	8/I	≥8/R	≥8/R	≥16/R	≥16/R	16/S	8/I	2/R	POS
Kp 03	*bla_KPC_* and *bla_NDM_*	≥32/R	≥128/R	≥64/R	≥64/R	≥64/R	4/I	≥8/R	≥8/R	≥16/R	≥16/R	4/S	≥16/R	2/R	POS
Kp 04	*bla_NDM_*	≥32/R	≥128/R	≥64/R	≥64/R	≥64/R	≥64/R	≥8/R	≥8/R	≥16/R	≥16/R	≤2/S	≥16/R	1/R	POS
Kp 05	*bla_KPC_*	≥32/R	≥128/R	≥64/R	≥64/R	≥64/R	≥64/R	≥8/R	≥8/R	≥16/R	≥16/R	≥64/R	4/S	≥4/R	POS
Kp 06	*bla_KPC_*	≥32/R	≥128/R	≥64/R	≥64/R	≥64/R	≤1/S	≥8/R	1/I	8/I	8/R	≤2/S	≤1/S	≤0.25/S	POS
Kp 07	*bla_KPC_* and *bla_NDM_*	≥32/R	≥128/R	≥64/R	≥64/R	≥64/R	≥64/R	≥8/R	≥8/R	≥16/R	≥16/R	32/I	≥16/R	≥4/R	POS
Kp 08	*bla_NDM_*	≥32/R	≥128/R	≥64/R	≥64/R	≥64/R	≥64/R	≥8/R	≥8/R	≥16/R	≥16/R	16/S	8/I	≥4/R	POS
Kp 09	*bla_NDM_,* and *bla_VIM_* and/or *bla_IMP_*	≥32/R	≥128/R	8/S	8/I	≥64/R	32/R	0.25/S	≤0.5/S	≥16/R	0.5/S	16/S	4/S	≤0.25/S	POS
Kp 10	*bla_KPC_* and *bla_NDM_*	≥32/R	≥128/R	≥64/R	≥64/R	≥64/R	≥64/R	≥8/R	≥8/R	≥16/R	≥16/R	16/S	8/I	≥4/R	POS
Kp 11	*bla_KPC_* and *bla_NDM_*	≥32/R	≥128/R	≥64/R	≥64/R	≥64/R	≥64/R	≥8/R	≥8/R	≥16/R	≥16/R	8/S	≥16/R	2/R	POS
Kp 12	*bla_KPC_, bla_NDM_,* and *bla_VIM_* and/or *bla_IMP_*	≥32/R	≥128/R	≥64/R	≥64/R	≥64/R	≥64/R	≥8/R	≥8/R	8/I	≥16/R	16/S	≤1/S	≤0.25/S	POS
Kp 13	*bla_KPC_* and *bla_NDM_*	≥32/R	≥128/R	≥64/R	≥64/R	≥64/R	≥64/R	≥8/R	≥8/R	≥16/R	≥16/R	8/S	≤1/S	≤0.25/S	POS
Kp 14	*bla_NDM_*	≥32/R	≥128/R	≥64/R	≥64/R	≥64/R	≥64/R	≥8/R	≥8/R	8/I	≥16/R	16/S	≥16/R	≥4/R	POS
Kp 15	*bla_KPC_* and *bla_NDM_*	≥32/R	≥128/R	≥64/R	≥64/R	≥64/R	32/R	≥8/R	≥8/R	≥16/R	8/R	32/I	≥16/R	≥4/R	POS
Kp 16	*NEG*	4/S	≤4/S	≤4/S	≤1/S	≤1/S	≤1/S	≤0.12/S	≤0.5/S	≤0.25/S	≤0.25/S	≤2/S	≤1/S	≤0.25/S	NEG
Kp 17	NEG	8/S	≤4/S	≤4/S	≤1/S	≤1/S	≤1/S	≤0.12/S	≤0.5/S	≤0.25/S	≤0.25/S	≤2/S	≤1/S	≤0.25/S	NEG
Kp 18	NEG	4/S	≤4/S	≤4/S	≤1/S	≤1/S	≤1/S	≤0.12/S	≤0.5/S	≤0.25/S	≤0.25/S	≤2/S	≤1/S	≤0.25/S	NEG
Kp 19	NEG	4/S	≤4/S	≤4/S	≤1/S	≤1/S	≤1/S	≤0.12/S	≤0.5/S	≤0.25/S	≤0.25/S	≤2/S	≤1/S	≤0.25/S	NEG
Kp 20	NEG	4/S	≤4/S	≤4/S	≤1/S	≤1/S	≤1/S	≤0.12/S	≤0.5/S	≤0.25/S	≤0.25/S	≤2/S	≤1/S	≤0.25/S	NEG
Kp 21	*NEG*	8/S	≤4/S	≤4/S	≤1/S	≤1/S	≤1/S	≤0.12/S	≤0.5/S	≤0.25/S	≤0.25/S	≤2/S	≤1/S	≤0.25/S	NEG
Kp 22	NEG	8/S	≤4/S	≤4/S	≤1/S	≤1/S	≤1/S	≤0.12/S	≤0.5/S	≤0.25/S	≤0.25/S	≤2/S	≤1/S	≤0.25/S	NEG
Kp 23	NEG	4/S	≤4/S	≤4/S	≤1/S	≤1/S	≤1/S	≤0.12/S	≤0.5/S	0.5/S	≤0.25/S	≤2/S	≤1/S	≤0.25/S	NEG
Kp 24	NEG	8/S	≤4/S	≤4/S	≤1/S	≤1/S	≤1/S	≤0.12/S	≤0.5/S	≤0.25/S	≤0.25/S	≤2/S	≤1/S	≤0.25/S	NEG
Kp 25	NEG	4/S	≤4/S	≤4/S	≤1/S	≤1/S	≤1/S	≤0.12/S	≤0.5/S	≤0.25/S	≤0.25/S	≤2/S	≤1/S	≤0.25/S	NEG
Kp 26	*NEG*	4/S	≤4/S	≤4/S	≤1/S	≤1/S	≤1/S	≤0.12/S	≤0.5/S	≤0.25/S	≤0.25/S	≤2/S	≤1/S	≤0.25/S	NEG
Kp 27	NEG	4/S	≤4/S	≤4/S	≤1/S	≤1/S	≤1/S	≤0.12/S	≤0.5/S	≤0.25/S	≤0.25/S	≤2/S	≤1/S	≤0.25/S	NEG
Kp 28	NEG	4/S	≤4/S	≤4/S	≤1/S	≤1/S	≤1/S	≤0.12/S	≤0.5/S	≤0.25/S	≤0.25/S	≤2/S	≤1/S	≤0.25/S	NEG
Kp 29	NEG	4/S	≤4/S	≤4/S	≤1/S	≤1/S	≤1/S	≤0.12/S	≤0.5/S	≤0.25/S	≤0.25/S	≤2/S	≤1/S	≤0.25/S	NEG
Kp 30	NEG	4/S	≤4/S	≤4/S	≤1/S	≤1/S	≤1/S	≤0.12/S	≤0.5/S	≤0.25/S	≤0.25/S	≤2/S	≤1/S	≤0.25/S	NEG
Kp ATCC 2146	*bla_NDM_*	≥32/R	≥128/R	≥64/R	≥64/R	≥64/R	≥64/R	≥8/R	≥8/R	≥16/R	≥16/R	≥64/R	≥16/R	≥4/R	NEG
Ec ATCC 25922	NEG	≤2/S	≤4/S	≤4/S	≤1/S	≤1/S	≤1/S	≤0.12/S	≤0.5/S	≤0.25/S	≤0.25/S	≤2/S	≤1/S	≤0.25/S	NEG

Abbreviations: MIC, minimal inhibitory concentration; SAM, ampicillin/sulbactam; TZP, piperacillin/tazobactam; FOX, cefoxitin; CAZ, ceftazidime; CRO, ceftriaxone; FEP, cefepime; DOR, doripenem; ETP, ertapenem; IPM, imipenem; MEM, meropenem; AMK, amikacin; GEN, gentamicin; CIP, ciprofloxacin; Kp, *K. pneumoniae;* R, resistant; I, intermediate; S, susceptible; NEG, negative; POS, positive. ^1^ Penicillin + β-lactamase inhibitors; ^2^ Cephamycins; ^3^ Extended-spectrum cephalosporins; ^4^ Carbapenems; ^5^ Aminoglycosides; ^6^ Fluoroquinolones.

**Table 2:** Antimicrobial susceptibility and resistance profiles of wild-type and multidrug-resistant strains of *P. aeruginosa.*

Strain ID	Carbapenemase Gene	MIC (μg/mL) for Antibiotic/Interpretative Categories										Carba NP Result
		TZP ^1^	CAZ ^2^	FEP ^2^	DOR ^3^	IPM ^3^	MEM ^3^	AMK ^4^	GEN ^4^	CIP ^5^	CST ^6^	
Pa 01	*bla_VIM_* and/or *bla_IMP_*	64/R	32/R	4/S	≥8/R	≥16/R	8/R	32/I	8/I	2/R	≤0.5/S	POS
Pa 02	*bla_NDM_, bla_VIM_* and *bla_IMP_*	≥128/R	≥64/R	32/R	≥8/R	≥16/R	≥16/R	≥64/R	≥16/R	≥4/R	≥16/R	POS
Pa 03	*bla_NDM_, bla_VIM_* and *bla_IMP_*	≥128/R	≥64/R	32/R	≥8/R	≥16/R	4/I	≥64/R	≥16/R	2/R	≤0.5/S	POS
Pa 04	*bla_VIM_* and *bla_IMP_*	≥128/R	32/R	32/R	≥8/R	≥16/R	8/R	≥64/R	4/S	≥4/R	≥16/R	POS
Pa 05	*bla_KPC_, bla_NDM_,* and *bla_VIM_* and/or *bla_IMP_*	≥128/R	≥64/R	16/I	≥8/R	≥16/R	8/R	≥64/R	4/S	2/R	≥16/R	POS
Pa 06	*bla_NDM_,* and *bla_VIM_* and/or *bla_IMP_*	≥128/R	≥64/R	32/R	≥8/R	≥16/R	≥16/R	≥64/R	8/I	≥4/R	≥16/R	POS
Pa 07	*bla_VIM_* and/or *bla_IMP_*	≥128/R	32/R	≤1/S	≥8/R	≥16/R	8/R	≥64/R	≥16/R	≥4/R	≥32/R	POS
Pa 08	*bla_VIM_* and/or *bla_IMP_*	≥128/R	≥64/R	≥64/R	≥8/R	≥16/R	≥16/R	≥64/R	≤1/S	≥4/R	≤0.5/S	POS
Pa 09	*bla_VIM_* and/or *bla_IMP_*	≥128/R	≥64/R	32/R	≥8/R	≥16/R	≥16/R	≥64/R	8/I	≥4/R	≥16/R	POS
Pa 10	*bla_VIM_* and/or *bla_IMP_*	≥128/R	≥64/R	32/R	≥8/R	≥16/R	≥16/R	≥64/R	≥16/R	≥4/R	≤0.5/S	POS
Pa 11	*bla_NDM_,* and *bla_VIM_* and/or *bla_IMP_*	≥128/R	≥64/R	≥64/R	≥8/R	≥16/R	≥16/R	8/S	4/S	0.5/S	≥16/R	POS
Pa 12	*bla_KPC_, bla_NDM_,* and *bla_VIM_* and/or *bla_IMP_*	≥128/R	32/R	16/I	≥8/R	≥16/R	4/I	32/I	≥16/R	≥4/R	≤0.5/S	POS
Pa 13	*bla_VIM_* and/or *bla_IMP_*	≥128/R	≥64/R	32/R	≥8/R	≥16/R	≥16/R	≥64/R	≥16/R	≥4/R	≥16/R	POS
Pa 14	*bla_VIM_* and/or *bla_IMP_*	≥128/R	≥64/R	≥64/R	4/I	1/S	4/I	≥64/R	≥16/R	≥4/R	≤0.5/S	POS
Pa 15	*bla_NDM_,* and *bla_VIM_* and/or *bla_IMP_*	≥128/R	≥64/R	16/I	≥8/R	≥16/R	≥16/R	≥64/R	≥16/R	≥4/R	≥16/R	POS
Pa 16	NEG	8/S	4/S	2/S	0.5/S	1/S	≤0.25/S	≤2/S	≤1/S	≤0.25/S	≤0.5/S	NEG
Pa 17	NEG	8/S	4/S	2/S	≤0.12/S	2/S	≤0.25/S	≤2/S	2/S	≤0.25/S	≤0.5/S	NEG
Pa 18	NEG	8/S	4/S	2/S	1/S	2/S	1/S	≤2/S	2/S	≤0.25/S	≤0.5/S	NEG
Pa 19	NEG	≤4/S	2/S	≤1/S	1/S	1/S	1/S	≤2/S	≤1/S	≤0.25/S	≤0.5/S	NEG
Pa 20	NEG	≤4/S	2/S	≤1/S	1/S	2/S	1/S	≤2/S	≤1/S	≤0.25/S	≤0.5/S	NEG
Pa 21	NEG	8/S	4/S	2/S	≤0.12/S	1/S	≤0.25/S	≤2/S	≤1/S	≤0.25/S	≤0.5/S	NEG
Pa 22	NEG	8/S	4/S	2/S	≤0.12/S	≤0.25/S	≤0.25/S	≤2/S	≤1/S	≤0.25/S	≤0.5/S	NEG
Pa 23	NEG	8/S	4/S	≤1/S	≤0.12/S	2/S	≤0.25/S	≤2/S	≤1/S	≤0.25/S	≤0.5/S	NEG
Pa 24	NEG	8/S	4/S	2/S	1/S	2/S	0.5/S	≤2/S	≤1/S	≤0.25/S	≤0.5/S	NEG
Pa 25	NEG	8/S	2/S	2/S	0.5/S	1/S	0.5/S	≤2/S	2/S	≤0.25/S	≤0.5/S	NEG
Pa 26	NEG	8/S	4/S	2/S	≤0.12/S	2/S	≤0.25/S	≤2/S	2/S	≤0.25/S	≤0.5/S	NEG
Pa 27	NEG	≤4/S	2/S	≤1/S	≤0.12/S	1/S	≤0.25/S	≤2/S	≤1/S	≤0.25/S	≤0.5/S	NEG
Pa 28	NEG	8/S	4/S	2/S	0.25/S	1/S	≤0.25/S	≤2/S	≤1/S	≤0.25/S	≤0.5/S	NEG
Pa 29	NEG	8/S	4/S	≤1/S	0.25/S	2/S	≤0.25/S	≤2/S	≤1/S	≤0.25/S	≤0.5/S	NEG
Pa 30	NEG	8/S	4/S	2/S	≤0.12/S	1/S	≤0.25/S	≤2/S	≤1/S	≤0.25/S	≤0.5/S	NEG
Pa ATCC 27853	NEG	≤4/S	≤1/S	≤1/S	0.25/S	2/S	≤0.25/S	≤2/S	≤1/S	≤0.25/S	≤0.5/S	NEG

Abbreviations: MIC, minimal inhibitory concentration; TZP, piperacillin/tazobactam; CAZ, ceftazidime; FEP, cefepime; DOR, doripenem; IPM, imipenem; MEM, meropenem; AMK, amikacin; GEN, gentamicin; CIP, ciprofloxacin; CST, listin; Pa, *P. aeruginosa;* R, resistant; I, intermediate; S, susceptible; NEG, negative; POS, positive. ^1^ Penicillin + β-lactamase inhibitors; ^2^ Extended-spectrum cephalosporins; ^3^ Carbapenems; ^4^ Aminoglycosides; ^5^ Fluoroquinolones; ^6^ Polymyxins.

**Table 3:** In vitro antibacterial and bactericidal activity of ΔM2 against wild-type and multidrug-resistant strains of *K. pneumoniae* and *P. aeruginosa*.

*K. pneumoniae* (*n*)	MIC (μg/mL)	MBC (μg/mL)	*P. aeruginosa* (*n*)	MIC (μg/mL)	MBC (μg/mL)	*p*-Value ^1^
WTKp (15)	4–8	4–8	WTPa (15)	8	8	0.1833
MDRKp (15)	8–16	8–16	MDRPa (15)	8–16	8–16	0.0016
Ec ATCC 25922	4–8	4–8	Pa ATCC 27853	4–8	4–8	
Kp ATCC 2146	8–16	8–16				

Abbreviations: MIC, minimal inhibitory concentration, MBC, minimal bactericidal concentration; WTKp, wild-type *K pneumoniae;* MDRKp, multidrug-resistant *K pneumoniae*; WTPa, wild-type *P aeruginosa*; MDRPa, multidrug-resistant *P aeruginosa*; Ec ATCC 25922, *Escherichia coli* ATCC 25922; Kp ATCC 2146, *K pneumoniae* ATCC 2146; Pa ATCC 27853, *P aeruginosa* ATCC 27853. ^1^ Significance level for the minimal inhibitory concentrations (MICs) between species, comparing independently wild-type strains and MDR strains.

**Table 4:** Tm, DH and FWHM values before and after adding the peptide to MLVs constituted by DMPC/DMPG (3:1).

Peptide-Lipid Molar Ratio	Pre-Transition Temperature (°C)	Tm ^1^ (°C)	DH ^2^ (cal/g)	FWHM ^3^ (°C)
0:1	13.55	22.98	0.32	0.55
1:10	NA	23.96	0.03	3.69

^1^ Temperature; ^2^ Enthalpy; ^3^ Full width at half maximum.
